# Evaluation of the safe use and dietary risk of *beta*-cypermethrin, pyriproxyfen, avermectin, diflubenzuron and chlorothalonil in button mushroom

**DOI:** 10.1038/s41598-017-07860-y

**Published:** 2017-08-18

**Authors:** Pengqiang Du, Xiaohu Wu, Hairong He, Ying Zhang, Jun Xu, Fengshou Dong, Yongquan Zheng, Xingang Liu

**Affiliations:** 1 State Key Laboratory for Biology of Plant Diseases and Insect Pests, Institute of Plant Protection, Chinese Academy of Agricultural Sciences, No. 2 Yuanmingyuan West Road, Haidian District, Beijing, 100193 China; 2 College of Chemistry, Central China Normal University, No. 152 Luoyu Road, Wuhan, 430079 China; 3 College of Life Science, Northeast Agricultural University, Mucai Street Xiangfang District No. 59, Harbin, 150030 China; 4 Guizhou Institute of Plant Protection, Guizhou, 55000 China

## Abstract

In this study, the residues of *beta*-cypermethrin, pyriproxyfen, avermectin, diflubenzuron and chlorothalonil in button mushrooms were investigated. The dietary risks of ingesting button mushrooms that have had these pesticides applied by two different methods under normal plant conditions were evaluated. The dissipation of these pesticides into the soil was also studied. According to the maximum residue limits (MRLs) and acceptable daily intakes (ADIs), the final residues of *beta*-cypermethrin, pyriproxyfen, avermectin, diflubenzuron, and chlorothalonil were safe for human consumption after these pesticides were applied by spraying 2 times at the dosages of 900, 750, 540, 562.5, and 540 g a.i.ha^−1^. The dissipation experiment results demonstrated that these pesticides dissipated rapidly after spraying, and there were no residues that could be detected at harvest time when the pesticides were mixed with substrates. According to this work, the application methods of spraying and incorporation with these pesticides at 1 and 1.5 times of the recommended dosage are safe and proper in cultivating button mushrooms.

## Introduction

*Agaricus bisporus* (button mushroom), a nutritious and environmentally friendly organic food, is rich in amino acids, chitin, vitamins and minerals and constitutes an increasing share in our diet^1, 2^. Button mushrooms exhibit antitumour activity, as demonstrated by various scientists and research groups^3–5^. The ability of button mushrooms to secrete extracellular enzymes to degrade cellulose, hemicellulose, lignin, protein and microbial cell wall polymers and produce small-molecular-weight compounds for assimilation and mycelial growth is environmentally friendly^6^. Given its nutritional value, medicinal value and environmentally friendly status, the button mushroom is widely cultivated worldwide. China has become the world’s leader in the cultivation of this species and produced over 2.18 million tons of button mushrooms in 2010^7^. More recently, production reached approximately 0.4 million tons and grossed approximately US $1.4 billion in the United States between 2012 and 2013^8^.

The cultivation of button mushrooms is subject to the same harm caused by pests and disease as are other plants. Specifically, dipteran pests regularly cause sizable economic losses worldwide. *Megaselia halterata* (Diptera: Phoridae) and *Lycoriella ingenua* (Diptera: Sciaridae) are the two prime insect pests in button mushroom cultivation^8, 9^. *Beta*-cypermethrin, pyriproxyfen, avermectin and diflubenzuron have the potential to control this pest. *Beta*-cypermethrin is a significant pesticide applied to control Diptera pests^10, 11^. Avermectin is a broad-spectrum pesticide that targets a number of pests, including Diptera, Lepidoptera, and Coleoptera^11, 12^. Avermectin also has the potential to control sciarid species. In Erler’s study, pyriproxyfen was useful in controlling Sciaridae on mushrooms and other dipteran pests^13, 14^. Diflubenzuron is an insect growth regulator and highly valuable in the control of sciarid species during mushroom cultivation^15–17^. Fungal pathogens, such as *Lecanicillium (Verticillium) fungicola, Mycogone perniciosa, Cladobotryum (Dactylium) spp*. and *Trichoderma spp*., can cause very serious fungal diseases in button mushrooms^18–20^, but chlorothalonil is a broad-spectrum fungicide that has the potential to control these diseases.

Spraying is a commonly used technique to apply pesticide. However, the pesticide cannot penetrate the substrate adequately through a spraying pattern alone. Kevin found that mushroom-growing substrates may attract the sciaridae^8^. The larvae feed on mycelia^16, 21^, compete with mycelia for nutrients^22^, disseminate mycoparasitic *Trichoderma* spp^23^, leave frass on the mycelia^24^ and reduce the water-holding capacity of the compost. Therefore, the incorporation of insecticides into mushroom compost is a significant benefit to mushroom cultivation^16^, and this method has been incorporated worldwide.

A large number of countries and international organizations have established strict pesticide residue limits in food and international trade rules to maintain food safety^25, 26^. These five pesticides have been frequently used on button mushrooms in China, but no study has reported on the final residue and dissipation dynamics of these pesticides in button mushroom cultivation to date. The aim of this study was to evaluate the dissipation rates of these pesticides through the button mushrooms growth environment and to determine the final residues on button mushrooms through different application methods to ensure that this produce is safe for human consumption.

## Results

### Linearity, recovery and detection limits

For soil samples, we employed a series of matrix-matched standard solutions (0.0005, 0.001, 0.005, 0.01, 0.05, 0.1, 0.5, 1, 5 mg L^−1^) for different pesticides by adding the blank soil extracting solution to each serially diluted standard solution to evaluate the linearity of the method. The lowest concentrations of *beta*-cypermethrin, pyriproxyfen, abamectin, diflubenzuron, and chlorothalonil were 0.01, 0.0005, 0.001, 0.001, and 0.01 mg L^−1^, respectively. As shown in Table 1, the correlation coefficients (R^2^) were greater than 0.998 for all pesticides, which confirms that the linearity was satisfactory. The limits of detection (LODs) for each of these compounds were estimated to be 0.016–1.5 mg kg^−1^, and the limits of quantification (LOQs) were 0.052–5.0 mg kg^−1^. The LOD was the concentration that produced a signal-to-noise (S/N) ratio of 3 compared with the low-fortified sample. The LOQ was defined based upon an S/N ratio of 10 compared with the low-fortified sample. To determine the matrix effect, the proposed method was determined by comparing the slope of the matrix-matched standards with the slope of the solvent standards. From Table 1, we can conclude that the matrix effect of abamectin was more obvious than the matrix effect for the other four pesticides after they were each extracted by the proposed preparation and detected by the equipment.

**Table 1:** Comparison of matrix-matched calibration and solvent calibration.

Compound	Matrix	Concentration (mg L^−1^)	Regression equation	R^2^	Slope of matrix/slope of solvent	LOD (mg kg^−1^)	LOQ (mg kg^−1^)
*beta*-cypermethrin	solvent	0.01–5	y = 8182.3x + 27.492	0.999	0.976	0.0016200	0.0054100
	soil	0.01–5	y = 7984.9x + 19.872	1.000			
pyriproxyfen	solvent	0.0005–5	y = 592254x + 57760	0.998	0.871	0.0000909	0.0003030
	soil	0.0005–5	y = 516082x + 32869	0.998			
abamectin	solvent	0.001–5	y = 32915x + 49.149	0.999	0.689	0.0001900	0.0006330
	soil	0.001–5	y = 22689x − 90.948	0.999			
diflubenzuron	solvent	0.001–5	y = 17032x + 404.55	0.999	0.888	0.0001650	0.0005500
	soil	0.001–5	y = 15125x − 338.61	0.999			
chlorothalonil	solvent	0.01–5	y = 14424x − 187.86	0.999	0.999	0.0019100	0.0063700
	soil	0.01–5	y = 14417x − 213.66	0.999			

The recoveries for each of these pesticides at different fortified levels were calculated over five replications (Table 2). The intra-day precision is represented as the standard deviation of the recoveries for a set of fortified samples on the same day. The inter-day precision was measured by the standard deviation of spiked samples over three distinct days. The data demonstrate that the recoveries were in the acceptable range of 69.5–109.2% for all pesticides at different levels. The intra-day precision and inter-day precision of the methods for repeatability achieved that requirement as they were 0.9–8.5% and 1.9–9.1%, respectively. The results of the recovery studies demonstrated that these preparation methods and detection procedures were also applicable for the residues of these pesticides in the soil.

**Table 2:** Recoveries (n = 5, %) and RSDs (%) for target compounds from soil.

	Spiked level (mg kg^−1^)	Recovery	RSD^a^	RSD^b^
*beta*-cypermethrin	0.01	75.2	2.5	3.6
	0.5	85.2	5.6	5.8
	5	89.8	2.8	1.9
pyriproxyfen	0.0005	76.8	1.9	3.8
	0.05	89.6	1.2	2.9
	5	102.5	6.5	4.1
abamectin	0.001	73.5	3.5	3.7
	0.01	95.2	4.5	8.4
	0.1	90.5	2.9	5.5
diflubenzuron	0.001	86.5	5.1	9.1
	0.05	105.2	0.9	6.5
	5	95.5	8.5	7.4
chlorothalonil	0.01	69.5	2.1	2.9
	0.5	109.2	4.2	2.5
	5	95.6	3.9	8.5

^a^Intra-day (n = 5). ^b^Inter-day (n = 15).

From the study of Du *et al*.^27^, the concentration ranges of *beta*-cypermethrin, pyriproxyfen, abamectin, diflubenzuron, and chlorothalonil were 0.01–5, 0.001–10, 0.001–10, 0.001–10, and 0.01–5 mg kg^−1^, respectively, with correlation coefficients greater than 0.998. The LODs of *beta*-cypermethrin, pyriproxyfen, abamectin, diflubenzuron, and chlorothalonil were 1.43, 0.333, 0.231, 0.176, and 1.00 mg kg^−1^, and the LOQs were 4.76, 1.00, 0.769, 0.588, and 3.33 mg kg^−1^, respectively.

### Dissipation of five pesticides in soil

**Figure 1:**
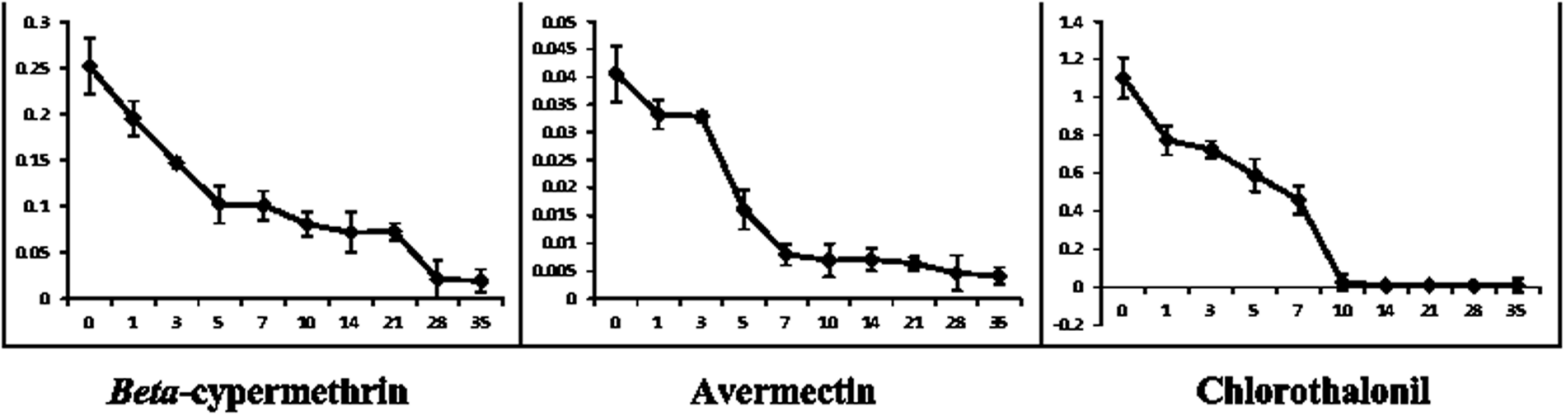
Dissipation patterns of *beta*-cypermethrin, avermectin and chlorothalonil in soil.

The dissipation of *beta*-cypermethrin, pyriproxyfen, avermectin, diflubenzuron and chlorothalonil was investigated in soil under the button mushrooms planting environment in Beijing. Figure 1 illustrates the dissipation curves for *beta*-cypermethrin, avermectin and chlorothalonil in soil. The initial concentration of *beta*-cypermethrin in the soil was 0.252 mg kg^−1^ with a half-life of 2.63 days. A sharp decrease in the amount of avermectin residue was noted in soil at less than 0.01 mg kg^−1^ in approximately 0–7 days with a half-life of 2.54 days. The initial concentration of chlorothalonil was 1.10 mg kg^−1^ with a half-life of 1.05 days, but less than 0.01 mg kg^−1^ chlorothalonil was noted 21 days after application. Pyriproxyfen residues were present at 2.21 mg kg^−1^ 2 h after application and 2.01 mg kg^−1^ 1 day after application, and the residues measured less than 0.1 mg kg^−1^ after 3 days. Diflubenzuron residues measured 1.2 mg kg^−1^ 2 h after application and were relatively low in the soil 1 day after application. The half-lives (t_1/2_), dissipation regressive equations, and correlation coefficients of *beta*-cypermethrin and avermectin are provided in Table 3. The values for pyriproxyfen and diflubenzuron were not calculated.

**Table 3:** Regression equation, correlation coefficient and half-lives of *beta*-cypermethrin, avermectin and chlorothalonil in soil.

Pesticides	Regression equation	Correlation coefficient (R^2^)	Half-life (days)
*beta*-cypermethrin	y = 0.3457e^−0.263x^	0.894	2.63
avermectin	y = 0.0508e^−0.273x^	0.912	2.53
chlorothalonil	y = 3.463e^−0.658x^	0.854	1.05

We also detected that the casing soil was influenced by the substrate mixed with pesticides, but no residues of any test pesticides were identified in the button mushrooms.

### Final residue analysis

Commercially formulated products utilizing these pesticides were sprayed after a few fruit bodies appeared, and the button mushrooms were collected after the application of the spray. The residues for pyriproxyfen and chlorothalonil were less than 0.001 and 0.01 mg kg^−1^ (their own low spike concentration), respectively. The residues of *beta*-cypermethrin, diflubenzuron and avermectin were less their respective low-spike concentration after 3 days of application at all dosages. The final residues from 2 h to 3 days are presented in Table 4. When sprayed at low dosages for 1 and 2 applications, the final residues of *beta*-cypermethrin, diflubenzuron, and avermectin were 0.0110–0.0921, 0.0812–0.0954, and 0.0123–0.0521 mg kg^−1^, respectively. When sprayed at high dosages over 1 and 2 applications, the final residues of *beta*-cypermethrin, diflubenzuron, and avermectin were 0.0193–0.102, 0.0804–0.161, and 0.0144–0.103 mg kg^−1^, respectively.

**Table 4:** Terminal residues of *beta*-cypermethrin, diflubenzuron and avermectin in button mushroom.

Pesticides	Days after spraying	Low dosage		High dosage	
		1 time	2 times	1 time	2 times
*beta*-cypermethrin	2 h	0.0452	0.0921	0.0369	0.102
	1 d	0.0327	0.0190	0.0252	0.0297
	3 d	0. 0110	0.0145	0.0231	0.0193
diflubenzuron	2 h	0.0954	0.0812	0.102	0.161
	1 d	<0.05	<0.05	<0.05	0.0804
	3 d	<0.05	<0.05	<0.05	<0.05
avermectin	2 h	0.0521	0.0452	0.0625	0.103
	1 d	0.0123	0.0134	0.0184	0.0259
	3 d	<0.005	<0.005	<0.005	0.0144

In the substrates mixed with the commercially formulated products, the button mushrooms were collected at harvest time, and the residues of the different pesticides at the different dosages were not detected in the button mushrooms.

### Acute dietary risk assessment

No residues were noted in the mushrooms when the pesticides were mixed with the substrate; therefore, the acute dietary risk was not considered in any further assessments.

According to Table 4, the residues of *beta*-cypermethrin, diflubenzuron, and avermectin were increased 2 h after spraying. Based on GB 2763–2014 in China, the dietary intake risks assessment for *beta*-cypermethrin, diflubenzuron and avermectin led to the ADIs of these chemicals being set at 0.02, 0.02 and 0.002 mg kg^−1^ body weight (bw), respectively. Assuming that the bw of the average adult is 60 kg in China, the ADIs for *beta*-cypermethrin, diflubenzuron and avermectin for each person are 1.2, 1.2 and 0.12 mg (ADIs of each chemical × 60 kg bw), respectively. According to the dietary guidelines in China^28^, the dietary intake for vegetables (including mushroom) is less than 0.35 kg each day. Based on the highest residue of these pesticides in mushrooms, the dietary intake of *beta*-cypermethrin, diflubenzuron and avermectin for each person would be 0.0357, 0.0564 and 0.0361 mg, respectively. The dosages of the pesticides are all less than their own recommended dosages.

## Discussion

The results of these experiments demonstrated that the residues of the pesticides were rapidly reduced in the soil. The residue of pyriproxyfen was less than 0.1 mg kg^−1^ 3 days after application. Diflubenzuron dissipated rapidly in soil with an estimated half-life of <1 day. The half-life of the chlorothalonil residue was 1.05 days. The half-lives of *beta*-cypermethrin and avermectin residues were slightly longer with half-lives of 2.63 and 2.53 days, respectively. The chlorothalonil concentration was less than 0.1 mg kg^−1^ in soil after application.

According to the final residue results, the final residues of pyriproxyfen and chlorothalonil in button mushrooms were relatively low, and the final residues of *beta*-cypermethrin, diflubenzuron, and avermectin were slightly higher 2 h after application. The residues of these pesticides were not detected in button mushrooms when the substrates were mixed with the recommended dosages. Compared with the MRLs of *beta*-cypermethrin, diflubenzuron, and chlorothalonil in China, the residues of the three pesticides were lower than their own MRLs in China. The MRLs of pyriproxyfen and avermectin in button mushrooms have not been established. Therefore, measurements of the *beta*-cypermethrin, diflubenzuron, and chlorothalonil residues suggested that they are safe when applied to button mushrooms according to the recommend dosages and recommended spraying times. The residues and ADIs of pyriproxyfen and avermectin will help administrators to establish the MRLs in button mushrooms. The spraying dose and spraying times will influence the residue content and pre-harvest interval^29, 30^. Adding the residues of the recommend dose and the 1.5 times recommended dose together, the measures for the residues of *beta*-cypermethrin, diflubenzuron, and avermectin were more influenced by the recommended spraying times.

In conclusion, the tested application methods with the tested dosages of *beta*-cypermethrin, pyriproxyfen, avermectin, diflubenzuron, and chlorothalonil in button mushrooms are safe to human and it might also be helpful for the establishment of MRLs of pyriproxyfen and avermectin in button mushrooms.

## Materials and Methods

### Materials

The certified reference standards of *beta*-cypermethrin (purity, 99%), pyriproxyfen (purity, 97%), avermectin (purity, 99.2%), diflubenzuron (purity, 99%) and chlorothalonil (purity, 99%) were provided by National Institute of Metrology (Beijing, China). *Beta*-cypermethrin 4.5% emulsifiable concentrate (EC) was obtained from the pesticide factory of the Institute of Plant Protection of Chinese Academy of Agricultural Sciences. Pyriproxyfen 10% suspension concentrate (SC) was obtained from Shanghai Bio-chemical and Agr-chemical Product Co., Ltd. Avermectin 5% EC was obtained from Hebei Veyong Bio-Chemical Co., Ltd. (Hebei, China). Diflubenzuron 20% SC was purchased from Hebei Veyong Bio-Chemical Co., Ltd. (Hebei, China), and chlorothalonil 75% water power (WP) was obtained from Limin Chemical Co., Ltd. (Jiangsu, China). The reagents and consumables have been mentioned in articles by Du *et al*.^27^.

A *beta*-cypermethrin standard solution of 100 mg L^−1^ was prepared in ethyl acetate and stored at 4 °C. A 100 mg L^−1^ standard solution of chlorothalonil was prepared in acetone and stored at 4 °C. For pyriproxyfen, diflubenzuron and avermectin, the standard stock solutions of 100 mg L^−1^ were prepared in chromatography grade methanol and stored at 4 °C, separately. *Beta*-cypermethrin calibration standard solutions were prepared in ethyl acetate and different matrixes extract from untreated samples at concentrations of 0.01–5.0 mg L^−1^. Pyriproxyfen, diflubenzuron and avermectin were prepared for standard curves by serial dilution at a 0.0005/0.001–5.0 mg L^−1^ concentration ranges and prepared in different matrixes extract from untreated samples at a 0.0005/0.001/0.005–5.0 mg L^−1^ concentration ranges.

### Substrate preparation, inoculation, incubation and harvest

Button mushrooms are grown on compost generally, and the compost is based on wheat straw, horse and chicken manure and other organic fertilizers in most countries. In our study, wheat straw (78%), chicken manure (20%) and slaked lime (2%) were adequately mixed. The chicken manure was dried and then ground into approximately 1-cm pieces, and the wheat straw was rolled and ground. This process increases its water-holder capacity, and the microbial activity was also increased to degrade proteins and polysaccharides^31^. At the composting stage, a layer of wheat was added, and that layer was 0.3 cm thick, 2 m long and 2 m wide. Then, a layer of manure that was just able to cover the wheat was added. The wheat and manure were alternately stacked until there were 12 layers. The temperature of heap was increased to 70–80 °C within 4–5 days. After the 6^th^ day, the temperature was decreased, and the compost was turned. From this point on, the compost was turned four times every 6 days. Slaked lime was added during the turning.

After the fourth turning, the compost was transferred to the mushroom house, and the compost was maintained at 20–25 cm thick. Other pesticides were used to control pests before the compost was transferred to the mushroom house. Then, 750 g of spawn (*A. bisporus* mycelium developed on cereal grain) per square metre was inoculated. In the incubation phase, the room temperature was less than 25 °C, and the relative humidity was maintained at 85–90%. The workshop air was well circulated. A week later, a non-nutritional layer of casing soil was added on top of the nutritious compost. The thickness of the soil was 3.5 cm, and the pH was adjusted to 7.5 (organic fertilizer, humic acid, and ammonium sulphate were added to decrease the pH; lime was added to increase the pH). At the harvest step, the room temperature was kept at 18–22 °C, and the relative humidity was maintained at 60–65%.

### Field experimental design

The field trials were performed in Taishitun, Miyun County, Beijing, and the trial was performed in a commercial button mushroom greenhouse equipped with an intelligent system to control temperature and humidity. This experiment was conducted in 2012, and the area of each trial plot measured 27 m^2^ with three replicated plots for each case. An isolation strip was utilized to prevent contamination from different plots in the same field. A trial plot without these pesticides was also implemented. During the trial, the average air temperatures were 13 °C, and the mean precipitation was 500–600 mm. The experimental environment was consistent with that of a greenhouse.

### Residue dynamic experiments

To investigate the dissipations of *beta*-cypermethrin, pyriproxyfen, avermectin, diflubenzuron and chlorothalonil in the soil, the button mushrooms were separately sprayed with the commercial products in the trial plots, each with three replicates. A one-time spray was performed at 1.5 times the recommended dosage. The dosage of *beta*-cypermethrin 4.5% EC, pyriproxyfen 10% SC, avermectin 5% EC, diflubenzuron 20% SC and chlorothalonil 75% WP were 900, 750, 540, 562.5, and 540 g a.i.ha^−1^, respectively. A plot of the same size but with no pesticide application was cultivated simultaneously. Soil was collected randomly from sampling plots at 2 h (0.0833 day, after spraying) and 1, 3, 7, 10, 14, 21, 28 and 35 days after the treatment. After harvesting, the samples were placed into polyethylene bags and immediately stored at −20 °C.

### Final residue experiments

To investigate the final residues of *beta*-cypermethrin, pyriproxyfen, avermectin, diflubenzuron and chlorothalonil in button mushrooms, the mushrooms were sprayed with the commercial products at two dosages (1 and 1.5 times of the recommended dosage) with three replicates, and these solutions were used to spray the remaining plots 1 to 2 times. The mushrooms were sprayed after a few fruit bodies appeared at the first flush, and the number of pests generally increased. The dosages of *beta*-cypermethrin 4.5% EC were 600 and 900 g a.i.ha^−1^. The dosages of pyriproxyfen 10% SC were 501 and 750 g a.i.ha^−1^. The dosages of avermectin 5% EC were 360 and 540 g a.i.ha^−1^. The dosages of diflubenzuron 20% SC were 375 and 562.5 g a.i.ha^−1^, and the dosages of chlorothalonil 75% WP were 360 and 540 g a.i.ha^−1^. The representative button mushroom samples were collected randomly from sampling plots at 2 h (0.0833 day, after spraying) and 1, 3, 7, 10, 14, 21, 28 and 35 days after the treatment. After harvesting, the samples were placed in polyethylene bags and immediately stored at −20 °C.

In addition, the final residues of these pesticides in button mushrooms that were applied by another approach were also investigated. Three level dosages of these pesticides were mixed with substrate with three replicates, and the pesticides were mixed before the substrate was transferred to the mushroom house. The dosages of *beta*-cypermethrin 4.5% EC mixed by substrate were 66.7, 200, and 600 mg per kilogram substrates. The dosages of pyriproxyfen 10% SC mixed by substrate were 56, 167, and 500 mg per kilogram substrates. The dosages of avermectin 5% EC mixed by substrate were 40, 120, and 360 mg per kilogram substrates. The dosages of diflubenzuron 20% SC mixed by substrate were 41.5, 125, and 375 mg per kilogram substrates. The dosages of chlorothalonil 75% WP were 333.3, 1000, and 3000 mg per kilogram substrates. Samples were collected at days 1, 3, 5, 7 and 14 when the fruit body began to take shape at the first flush. After picking, the samples were put into polyethylene bags and kept at −20 °C, immediately.

### Analytical methods

Ten grams (10 g) of homogenized button mushrooms were weighed in a tube, and 2.0 ml of water and 10.0 ml of organic solvent (acetonitrile for pyriproxyfen, abamectin, and diflubenzuron; ethyl acetate for *beta*-cypermethrin and chlorothalonil) were then added to extract the pesticides from homogenized mushrooms. Then, NaCl and anhydrous MgSO_4_ were added and vortexed for 1 min and then centrifuged for 5 min. One and a half millilitres (1.5 ml) of the supernatant was transferred into a centrifuge tube containing 100 mg of anhydrous MgSO_4_ and 50 mg of purificant (primary-secondary amine for pyriproxyfen, abamectin, and diflubenzuron; florisil for *beta*-cypermethrin and chlorothalonil). The supernatant was filtered with 0.22-mm filters for detection. For a full description, see Du *et al*.^27^.

### Statistical analysis

The dissipation curves of these pesticides were calculated using nonlinear regression with Microsoft Excel software. The degradation kinetics followed first-order kinetics C = C_0_e^−kt^, where C represents the concentration (mg kg^−1^) of the chemical at time t (days), C_0_ represents the initial concentration (mg kg^−1^), and k is the first-order rate constant (day^−1^) independent of C and C_0_. The correlation coefficient is used to represent the congruence between the data and the first-order dynamic equation. The half-life is defined as the time required for the pesticide residue level to be reduced to 50% of the initial residue level after application and can be calculated using the Hoskins formula (DT50 = (ln 2)/k).
